# Proteome-Wide Analysis of Lysine 2-Hydroxyisobutyrylation in *Aspergillus niger* in Peanuts

**DOI:** 10.3389/fmicb.2021.719337

**Published:** 2021-08-18

**Authors:** Manlin Xu, Xia Zhang, Jing Yu, Zhiqing Guo, Ying Li, Xinying Song, Kang He, Guowei Li, Yucheng Chi

**Affiliations:** ^1^Shandong Peanut Research Institute, Qingdao, China; ^2^Institute of Crop Germplasm Resources, SAAS, Jinan, China

**Keywords:** *Aspergillus niger*, proteome, post-translational modifications, pathogenesis, lysine 2-hydroxyisobutyrylation

## Abstract

*Aspergillus niger* is a very destructive pathogen causing severe peanut root rot, especially in the seeding stage of peanuts (*Arachis hypogaea*), and often leading to the death of the plant. Protein lysine 2-hydroxyisobutyrylation (Khib) is a newly detected post-translational modification identified in several species. In this study, we identified 5041 Khib sites on 1,453 modified proteins in *A. niger*. Compared with five other species, *A. niger* has conserved and novel proteins. Bioinformatics analysis showed that Khib proteins are widely distributed in *A. niger* and are involved in many biological processes. Gene Ontology (GO) and Kyoto Encyclopedia of Genes and Genomes (KEGG) enrichment analyses revealed that Khib proteins were significantly enriched in many cellular compartments and pathways, such as ribosomes and proteasome subunits. A total of 223 Khib proteins were part of the PPI network, thus, suggesting that Khib proteins are associated with a large range of protein interactions and diverse pathways in the life processes of *A. niger*. Several identified proteins are involved in pathogenesis regulation. Our research provides the first comprehensive report of Khib and an extensive database for potential functional studies on Khib proteins in this economically important fungus.

## Introduction

Protein post-translational modifications (PTMs) are important regulatory mechanisms that introduce new functionalities and dynamically control protein activity by modulating intra- and intermolecular interactions, thus, enhancing protein capabilities. PTMs are involved in almost all living cells and all aspects of cellular processes, including protein synthesis, assembly, localization, function, and degradation (Müller, 2018). To quickly respond to changes in the environment and address environmental stress, PTMs occur much faster than new protein synthesis and are also necessary for new protein synthesis. PTMs enable living organisms to adapt and survive in changing environments; are involved in key biological pathways in the fine regulation of multiple physiological processes in cells; and are closely associated with the occurrence and development of all types of disease (Lin and Caroll, 2018). To date, most identified PTMs occur in the form of reversible modifications of amino acid residues, frequently lysine (Boussouar et al., 2008; Chen et al., 2009; Yuan et al., 2014). With the development of high-throughput technologies such as mass spectrometry (MS), increasing numbers of modifications have been identified and functionally analyzed (Yuan et al., 2014), including ubiquitylation, phosphorylation, lysine acylation, crotonylation, succinylation, malonylation, β-hydroxybutyrylation, and 2-hydroxyisobutyrylation (Allfrey et al., 1964; Tan et al., 2011; Xie et al., 2012, 2016; Medina and Avila, 2015; Meyer et al., 2016; Nandakumar et al., 2021). These studies have revealed that newly identified modifications have specific effects on cell growth, differentiation, metabolism, and other life processes.

Lysine 2-hydroxyisobutyrylation was first reported in 2014, in a study identifying the mark at 63 human and mouse histone Khib sites, 27 of which were unique lysine sites not modified by lysine acetylation (Kac) and lysine crotonylation (Kcr) (Dai et al., 2014). During male germ cell differentiation, histone Khib shows genomic distributions different from those of Kac and Kcr. Furthermore, the histone Khib mark has high stoichiometry and substantial structural changes, and is conserved and widely distributed (Dai et al., 2014). The amount of H4K8 Khib changes in response to the carbon source availability in *Saccharomyces cerevisiae*, and the medication is diminished in low-glucose conditions. In agreement with proteomic analysis results, large amounts of 2-hydroxyisobutyrylated proteins have been found to take part in glycolysis/gluconeogenesis. Thus, the Khib mark not only regulates chromatin functions but also forms a network linking Khib, glucose metabolism and CLS (Huang et al., 2017). To date, Khib has been reported in several species, as described in the first report of global profiling of the Khib proteome in mammalian cells, which discovered both the “writers” and “erasers” of histone Khib marks and major Khib protein substrates (Huang et al., 2018). Large amounts of Khib have been reported in plants, such as developing rice seeds (Meng et al., 2017), common wheat (Bo et al., 2021; Zhang et al., 2021) and *Physcomitrella patens* (Yu et al., 2017); Histone H4K8 2-hydroxyisobutyrylation (H4K8 hib) has been identified as an evolutionarily conserved modification in *Saccharomyces cerevisiae* (Huang et al., 2017). Some reports have described Khib in biological pathogens, such as the animal pathogens *Proteus mirabilis*, *Toxoplasma gondii*, and *Candida albicans* (Dong et al., 2018; Yin et al., 2019; Zheng et al., 2021), Khib in plant pathogens has also been reported, such as *Fusarium oxysporum*, *Botrytis cinerea*, and *Ustilaginoidea virens* (Xu et al., 2020; Chen et al., 2021; Qian et al., 2021). Reports on these three pathogens have revealed that Khib is widely distributed in cellular compartments and is involved in diverse cellular processes, including regulation of pathogenicity and virulence.

*Aspergillus niger*, a member of the black *Aspergilli*, is infectious throughout the entire peanut growth period, especially in the seeding and early growth stages. It mainly infects the stem base, directly causing seeding death and resulting in large losses in peanut production. The genome of *A. niger* is approximately 33 Mb, arranged in 19 supercontigs. Among the predicted 14,000 proteins, approximately half are associated with metabolism, cellular transport, and protein fate (Pel et al., 2007). Few reports have described PTMs, and no report has described Khib, in *A. niger*. To investigate the mechanisms of *A. niger* and their influence on cell function, we performed a proteome-wide analysis of Khib in *A. niger* in peanuts and identified 5041 Khib sites on 1,453 modified proteins in *A. niger*. These identified proteins are widely distributed in *A. niger* cells and are involved in many biological processes. We analyzed the Khib site motif and the modified protein structure; compared the conservation of the identified proteins with those in other species; used Gene Ontology (GO) and Kyoto Encyclopedia of Genes and Genomes (KEGG) to analyze the proteins’ functions, pathways, cellular localization and domain enrichment; investigated the protein relationship on the basis of protein-protein interaction (PPI) network analysis; and summarized the proteins associated with pathogenicity.

This work provides insights into Khib in *A. niger* and explores the function of the identified proteins in this economically important fungus.

## Materials and Methods

### Fungal Strain and Culture

The *A. niger* strain LXGF was isolated from peanut root rot. Its cultural characteristics and molecular identification confirmed that the fungus isolated from the infected peanut samples was *A. niger*. The fungi were inoculated on potato dextrose agar medium and cultured under 25°C in the dark for 3 days. The conidia and mycelia were collected and immediately frozen in liquid nitrogen and then stored at −80°C.

### Protein Extraction and Trypsin Digestion

The mycelia and spores were ground into powder in liquid nitrogen, then sonicated three times on ice with a high intensity ultrasonic processor (Scientz, Ningbo, China) in lysis buffer (8 M urea, 2 mM EDTA, 3 μM TSA, 50 mM NAM, 10 mM DTT and 1% protease inhibitor cocktail). The remaining debris was removed by centrifugation at 20,000 *g* at 4°C for 10 min. Finally, the protein was precipitated with cold 15% TCA for 2 h at −20°C. After centrifugation at 4°C for 10 min, the supernatant was discarded. The remaining precipitate was washed with cold acetone three times. The protein was redissolved in buffer (8 M urea and 100 mM NH_4_CO_3_, pH 8.0), and the protein concentration was determined with a 2-D Quant kit (GE Healthcare, Beijing, China) according to the manufacturer’s instructions. For digestion, the protein solution was reduced with 10 mM DTT for 1 h at 37°C and alkylated with 20 mM IAA for 45 min at room temperature in the dark. For trypsin digestion, the protein sample was diluted by addition of 100 mM NH_4_CO_3_ to a urea concentration less than 2 M. Finally, trypsin was added at a 1:50 trypsin-to-protein mass ratio for the first digestion overnight and a 1:100 trypsin-to-protein mass ratio for a second 4 h-digestion.

### HPLC Fractionation

The sample was then fractionated with high pH reverse-phase HPLC with an Agilent 300Extend (Agilent, Shanghai, China) C18 column (5 μm particles, 4.6 mm ID, 250 mm length). Briefly, peptides were first separated into 80 fractions with a gradient of 2 to 60% acetonitrile in 10 mM ammonium bicarbonate, pH 10, over 80 min. Then, the peptides were combined into six fractions and dried by vacuum centrifugation.

### Affinity Enrichment

To enrich peptides, we incubated tryptic peptides dissolved in NETN buffer (100 mM NaCl, 1 mM EDTA, 50 mM Tris–HCl, and 0.5% NP-40, pH 8.0) with pre-washed agarose-conjugated anti Khib antibody beads (Micrometer Biotech, Hangzhou, China) at 4°C overnight with gentle shaking (Bo et al., 2021). The beads were washed four times with NETN buffer and twice with ddH_2_O. The bound peptides were eluted from the beads with 0.1% TFA. The eluted fractions were combined and vacuum-dried. The resulting peptides were cleaned with C18 ZipTips (Millipore, Billerica, MA, United States) according to the manufacturer’s instructions, and this was followed by LC-MS/MS analysis.

### LC-MS/MS Analysis

The Khib peptides were dissolved in 0.1% FA (Fluka, Shanghai, China) and directly loaded onto a reversed-phase pre-column (Acclaim PepMap 100, Thermo Scientific, Waltham, MA, United States), Peptide separation was performed with a reversed-phase analytical column (Acclaim PepMap RSLC, Thermo Scientific, Waltham, MA, United States). The gradient comprised an increase from 6 to 22% solvent B (0.1% FA in 98% ACN) for 24 min; 22 to 40% for 8 min; an increase to 80% in 5 min; and a hold at 80% for the last 3 min, all at a constant flow rate of 300 nl/min on an EASY-nLC 1000 UPLC system. The resulting peptides were analyzed with a Q Exactive^TM^ Plus hybrid quadrupole-Orbitrap mass spectrometer (Thermo Fisher Scientific, Waltham, MA, United States).

The peptides were subjected to NSI source followed by tandem mass spectrometry (MS/MS) in Q Exactive^TM^ plus (Thermo Scientific, Waltham, MA, United States) coupled online to UPLC. Intact peptides were detected in the Orbitrap at a resolution of 70,000. Peptides were selected for MS/MS with an NCE setting at 30; ion fragments were detected in the Orbitrap at a resolution of 17,500. A data-dependent procedure that alternated between one MS scan and 20 MS/MS scans was applied for the top 20 precursor ions above a threshold ion count of 5E3 in the MS survey scan with 15.0 s dynamic exclusion. The electrospray voltage was 2.0 kV. Automatic gain control was used to prevent overfilling of the orbitrap; 5E4 ions were accumulated for generation of MS/MS spectra. For MS scans, the m/z scan range was 350 to 1,800. The fixed first mass was set as 100 *m/z*. LC–MS/MS analysis was carried out by Micrometer Biotech Company (Hangzhou, China).

### Database Search

The resulting MS/MS data were processed using MaxQuant, with the integrated Andromeda search engine (v.1.5.2.8). Tandem mass spectra were searched against *Aspergillus niger* fasta (*A. niger* sequencing results identified 26,472 total proteins) concatenated with the reverse decoy database. Trypsin/P was specified as the cleavage enzyme, and as many as four missing cleavages, five modifications per peptide and five charges were allowed. The mass error was set to 10 ppm for precursor ions and 0.02 Da for fragment ions. Carbamido methylation on cysteine was specified as a fixed modification, and oxidation of methionine, crotonylation of lysine and acetylation of protein N-termini were specified as variable modifications. thresholds for protein, peptide and modification sites were specified at 1% (Elias and Gygi, 2007). The minimum peptide length was set at 7. All other parameters in MaxQuant were set to default values. The site localization probability was set as > 0.75.

### Bioinformatics Methods

The Soft MoMo (motif-x algorithm) was used to analyze the model of sequences comprising amino acids in specific positions of modified-21-mers (10 amino acids upstream and downstream of the site, but phosphorylation with modified-13-mers six amino acids upstream and downstream of the site) in all protein sequences. All database protein sequences were used as background database parameters. The minimum number of occurrences was set to 20. The emulate original motif-x was selected, and other parameters were set at default. We used GO, a major bioinformatics initiative to unify the representation of gene and gene product attributes across all species. The GO annotation proteome was derived from the UniProt-GOA database^1^. The KEGG database was used to annotate protein pathways, by connecting known information on molecular interaction networks. We used wolfpsort subcellular localization predication software to predict subcellular localization. Wolfpsort is an updated version of PSORT/PSORT II for the prediction of eukaryotic sequences. Identified protein domain functional descriptions were annotated with InterProScan^2^ on the basis of the protein sequence alignment method.

To determine the degree of evolutionary conservation of 2-hydroxyisobutyrylation, we first used BLASTP to compare 2-hydroxyisobutyrylated protein sequences of *A. niger* against specified 2-hydroxyisobutyrylated protein sequences. By applying a reciprocal best BLAST hit approach, we determined the orthologous proteins among these proteins. For each orthologous group, we used MUSCLE v3.8.31 for multiple sequence alignment (Edgar, 2004). *P*-values were calculated for each comparison with Fisher’s exact test. For all crotonylated proteins identified (which are associated with the amino acid metabolism process), name identifiers were searched against the STRING database version 10.0^3^ for PPI. Only interactions between the proteins belonging to the searched data set were selected, thereby excluding external candidates. STRING defines a metric called the “confidence score” to define interaction confidence; we selected all interactions that had a confidence score ≥ 0.9 (high confidence). The interaction network from STRING was visualized in Cytoscape (Version 3.3.0). A graph of the theoretical clustering algorithm molecular complex detection (MCODE) was used to analyze densely connected regions.

## Data Availability

The mass spectrometry proteomics data have been deposited in the ProteomeXchange, data are available via ProteomeXchange with identifier PXD026373.

## Results

### Identification of 2-Hydroxyisobutyrylated Proteins in *A. niger*

In the experimental procedures (Supplementary Figure 1A) used to identify Khib proteins and Khib sites in *A. niger*, three biological repeats were examined under the same experimental conditions. A total of 64,059 secondary mass spectrograms were obtained by mass spectrometry analysis. After protein theoretical data were searched, the available number of effective spectrograms was 40,804, and the utilization rate of spectrograms was 63.7%. Among all 8,919 detected peptides, 8,431 peptides (94.5%) had Khib modification. We identified a total of 8,471 sites on 2,030 proteins, and 1,453 proteins had 5,041 identical modification sites in three replicates (Supplementary Tables 1, 2). The lengths of most peptides were 7–20 amino acids, and the length of the distribution of the mass spectrometry identification to meet the sample quality requirements (Supplementary Figure 1B). The mass error of most spectrograms was within 10 ppm, because of the high-precision characteristics of orbital well mass spectrometry, thus, indicating that the mass accuracy of the mass spectrometer was normal, and the qualitative and quantitative analysis of proteins conformed to the standards (Supplementary Figure 1C). Most proteins had fewer than three modified sites, only a few more than seven Khib sites. The data are available via ProteomeXchange^4^ under ID number PXD026373. *A. niger* sequencing results revealed 26,472 total proteins in *A. niger* (Aspergillus niger. fasta), the identified 2-hydroxyisobutyrylome covered proteins of the proteome, account for 5.49% (1,453/26,472).

### Analysis of the Khib Site Motifs

**FIGURE 1:**
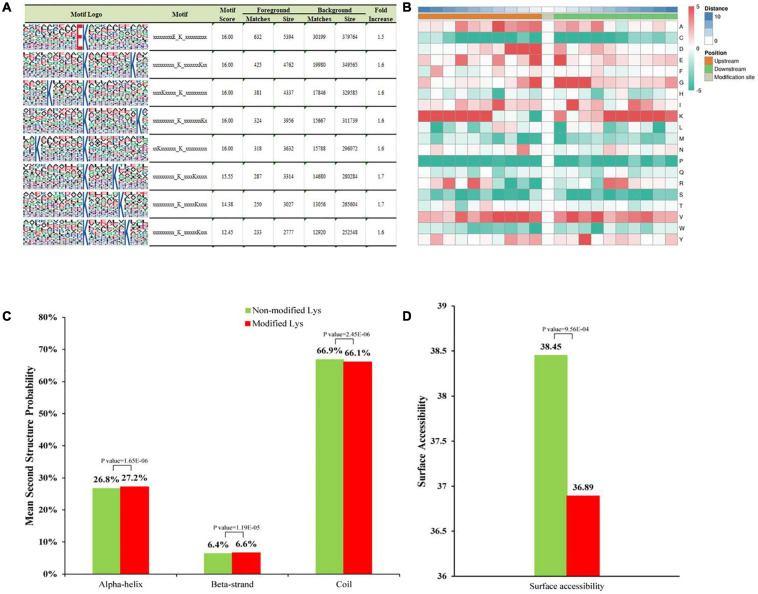
Properties of Khib peptides in *A. niger*. **(A)** Motif analysis shows Khib peptide motifs and conservation of Khib sites. The intensity map shows enrichment of amino acids in particular positions around the Khib lysine residues. **(B)** Heat map of the amino acid compositions around the Khib sites. Red indicates enrichment and green indicates depletion. **(C)** Probabilities of Khib in different protein secondary structures (alpha helix, beta-strand, and coil structures). **(D)** Predicted surface accessibility of Khib sites.

We used the MoMo program to evaluate specific amino acid sequence motifs around the Khib sites. We detected 16 conserved motifs in the peanut 2-hydroxysobutyrylome around Khib sites (Figure 1A and Supplementary Table 3). They were identified for 10 amino acids upstream and downstream of Khib sites (−10 Khib + 10) among 5,041 peptides, accounting for 83.2% (4,209/5,041) of all identified peptides. The EKhib, K(X5)Khib, K(X6)Khib and K(X7)Khib (X represents an unspecified amino acid residue), motifs were also found in other species, such as *Botrytis cinerea* and *Fusarium oxysporum*, thus indicating that they are conserved Khib modification motifs in different species. Heat-map analysis showed the following frequencies of amino acids flanking the Khib sites (Figure 1B): aspartate (D) at −1, −2, and −3 positions; glutamate (E) at −1 and + 1 positons; glycine (G) at −1, + 1, + 2 and + 3 positions; lysine (K) at the −10 to −5 and + 5 to + 10 positions; valine (V) at −4 to −2 and + 2, + 4 positions are favorable for 2-hydroxyisobutyrylation.

### Structural Analysis of All the 2-Hydroxyisobutyrylated Proteins

Secondary structure analysis was performed with NetSurfP to detect the preferred structure of the Khib sites in proteins. A total of 66.9% of the Khib sites were located in disordered coils, 26.8% of the sites were in α-helices, and the rest of the sites were in β-strands. This distributions between the 2-hydroxyisobutyrylated lysines and non-modified lysines were very similar (Figure 1C). Moreover, 2-hydroxyisobutyrylated sites were less surface-accessible than the corresponding unmodified regions (Figure 1D). The surface accessibly was higher in non-modified sites and lower in Khib sites, thus, indicating that the modification may occur selectively in biological processes.

### Conserved Analysis of Khib Proteins in Different Species

**FIGURE 2:**
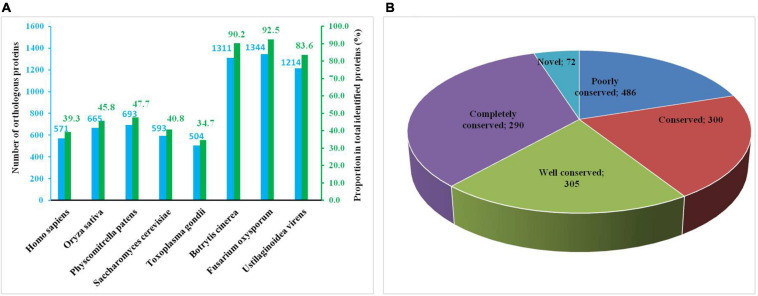
Conserved analysis of Khib proteins in different species. **(A)** Number of orthologous Khib proteins in five organisms with reported 2-hydroxyisobutyrylomes. **(B)** A pie chart of conservation of Khib proteins in five organisms. Grouping was performed as follows: Completely conserved, 5 orthologs; Well conserved, 4 orthologs; Conserved, 3 orthologs; Poorly conserved, 1 to 2 orthologs; Novel, 0 orthologs.

To determine the degree of evolutionary conservation of Khib, we first used BLASTP to compare 2-hydroxyisobutyrylated protein sequences of *A. niger* against specified 2-hydroxyisobutyrylated protein sequences, including eight species: *Homo sapiens*, *Physcomitrella patens*, *Oryza sativa subsp. japonica*, *Saccharomyces cerevisiae*, *Toxoplasma gondii, Botrytis cinerea*, *Fusarium oxysporum*, and *Ustilaginoidea virens*. By applying a reciprocal best BLAST hit approach, we determined the orthologous proteins among the searched proteins. In total, we identified 6,895 orthologs of the 2-hydroxyisobutyrylated proteins in *A. niger* in the eight species (Figure 2A and Supplementary Table 4). Among the 1,453 identified proteins in *A. niger*, the number of orthologous proteins of *Homo sapiens*, *Oryza sativa*, *Physcomitrella patens*, *Saccharomyces cerevisiae*, *Toxoplasma gondii*, *Botrytis cinerea*, *Fusarium oxysporum*, and *Ustilaginoidea virens* were 571, 665, 693, 593, 504, 1311, 1344, and 1214, accounted for 39.3, 45.8, 47.7, 40.8, 34.7, 90.2, 92.5, and 83.6%, respectively (Figure 2A). On the basis of the different species’ homologous proteins, 20.0% (290/1,453) of the proteins were found in eight species and were identified as completely conserved proteins, 21.0% (305/1,453) of the proteins were found in 6–7 species and were identified as well conserved proteins, 20.6% (300/1,453) of the proteins were found in 4–5 species and were identified as conserved proteins, 33.4% (486/1453) of the proteins were found in 1–3 species and were identified as poorly conserved proteins, and 5.0% (72/1,453) of the proteins were found in zero species and were identified as novel proteins (Figure 2B and Supplementary Table 4).

### Functional Annotation and Cellular Localization of Khib Proteins in *A. niger*

**FIGURE 3:**
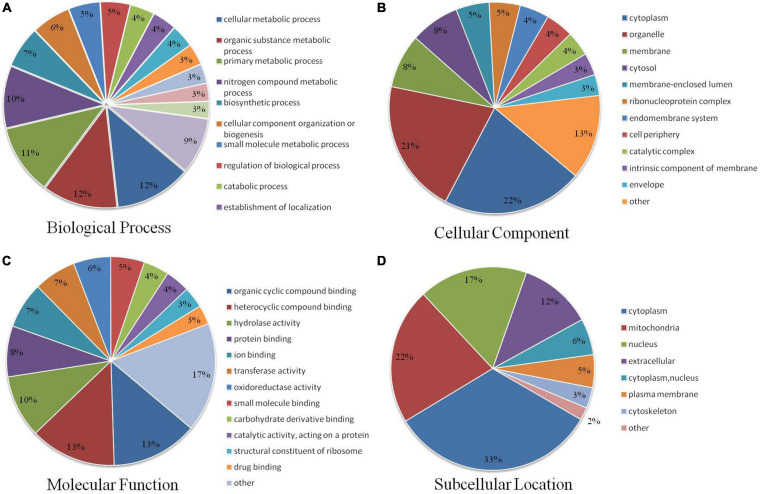
Pie charts showing the distribution of Khib proteins. **(A)** Khib proteins categorized according to biological process. **(B)** Cellular component. **(C)** Molecular function. **(D)** Subcellular localization.

To investigate the functions of Khib proteins in *A. niger*, we classified them into three categories – biological processes, cellular component and molecular function – through GO term classification analysis. In the biological processes, at the top of the list, with the largest number of proteins, were cellular metabolic, organic substance metabolic, primary metabolic process and nitrogen compound metabolic process, containing 702, 688, 642, and 573 identified proteins, respectively (Figure 3A and Supplementary Table 5). Together, the top four terms contained approximately 40–50% of the total identified proteins (1,453). Many Khib proteins were found to be involved in metabolic processes. According to the cellular component, there were 783, 744, 300, and 273 proteins in the cytoplasm, organelle, membrane, and cytosol, respectively (Figure 3B). Among them, Khib proteins in the cytoplasm and organelles contained approximately half the total identified proteins. Khib proteins were found to be distributed in the organelles and cell matrix. As for the molecular function categories, most Khib proteins were found to be involved in organic cyclic compound binding (274), heterocyclic compound binding (273), hydrolase activity (198), protein binding (161), ion binding (147), transferase activity (134), oxidoreductase activity (118), and small molecule binding (107) (Figure 3C). On the basis of GO analysis of Khib protein function, the identified proteins were found to be widely distributed in cells and organelles, and to be involved in different biological processes related to molecular function in *A. niger*.

Subcellular localization analysis of Khib proteins in *A. niger* showed that the identified proteins mainly localized in the cytoplasm, mitochondria, nucleus and outside the cell, with 480, 315, 252, and 169 identified proteins, respectively (Figure 3D and Supplementary Table 6), accounting for approximately 12–33% of the total identified proteins (1,453). A small number of identified proteins localized in the cytoplasm, nucleus, plasma membrane, cytoskeleton and other areas accounted for approximately 12–33% of the total identified proteins (1,453). These results revealed that Khib proteins have extensive biological functions and are widely distributed in *A. niger.*

### Function, Pathway and Domain Enrichment Analyses of Khib Proteins in *A. niger*

**FIGURE 4:**
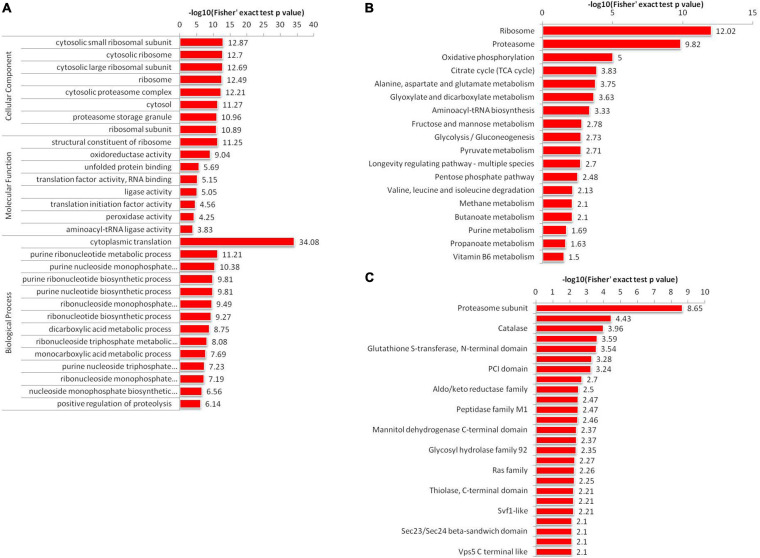
Enrichment analysis of 2-hydroxyisobutyrylated proteins in *A. niger*. **(A)** GO-based enrichment analysis of identified proteins. **(B)** KEGG pathway-based enrichment analysis. **(C)** Protein domain enrichment analysis of all identified proteins.

To further reveal the characteristics of Khib proteins in *A. niger*, we used GO, KEGG pathway and protein domains analysis. The GO enrichment revealed that in cellular component, large numbers of proteins were enriched in the cytosol and ribosomes (Figure 4A and Supplementary Table 7). According to the molecular function category, the proteins associated with the structural constituents of ribosome, oxidoreductase activity, unfolded protein binding, translation factor activity, RNA binding, ligase activity, translation initiation factor activity, peroxidase activity and aminoacyl-tRNA ligase activity were more likely to be 2-hydroxyisobutyrylated. In the biological process category, proteins involved in cytoplasmic translation, purine ribonucleotide metabolic process, purine ribonucleotide biosynthetic process, purine nucleotide biosynthetic process, ribonucleoside monophosphate metabolic process and ribonucleotide biosynthetic process were more likely to be 2-hydroxyisobutyrylated.

Kyoto Encyclopedia of Genes and Genomes pathway enrichment analysis showed that Khib proteins were significantly enriched in 18 pathways in *A. niger* (Figure 4B and Supplementary Table 8). Among all 18 enriched pathways, the top two enriched pathways were the ribosome (map03010) and proteasome (map03050). The results were corresponded to the GO analysis results. These two pathways were associated with protein synthesis and degradation. In addition, we identified another 16 enriched pathways, including several associated with protein synthesis and processing in cells, such as alanine, aspartate and glutamate metabolism (map00250), glyoxylate and dicarboxylate metabolism (map00630) and aminoacyl-tRNA biosynthesis (map00970). Other pathways were associated with energy production, such as oxidative phosphorylation (map00190), citrate cycle (TCA cycle) (map00020), glycolysis/Gluconeogenesis (map00010), fructose and mannose metabolism (map00051), valine, leucine and isoleucine degradation (map00280), Pentose phosphate pathway (map00030) and pyruvate metabolism (map00620).

Analysis of protein domain enrichment revealed many findings corresponding to the GO and KEGG results. The identified Khib proteins were enriched in 25 domain families (Figure 4C and Supplementary Table 9). The top enriched protein domain were proteasome subunit, proteasome subunit A N-terminal signature, catalase, ubiquitin family, glutathione S-transferase, N-terminal domain, Septin, and PCI domain. All the functional enrichment analyses suggested that Khib proteins were not only widely spread in *A. niger* but also play important roles in cell synthesis and metabolism.

### Khib Proteins Are Involved in Central Metabolism

From the KEGG pathway-based enrichment analysis indicated that Khib was associated with a large amount of proteins involved in ribosomes and proteasomes. As for ribosomes, both the large subunit and small unit proteins were nearly half Khib modified with several sites (Supplementary Figure 2A). The three domains of proteasomes complex showed multiple modified proteins (Supplementary Figure 2B), particularly, all the 14 subunits (from α1 to α7, β1 to β7) of the standard proteasome subunits be modified, they belong to the core particles (20S proteasome). These results suggested that Khib may play an critical roles in protein synthesis and degradation.

### Protein-Protein Interaction (PPI) Network of Khib Proteins in *A. niger*

**FIGURE 5:**
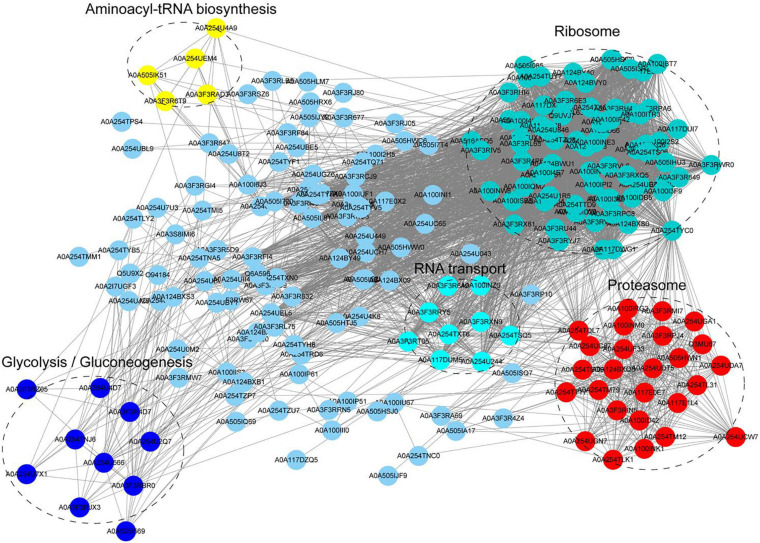
Protein-protein interaction (PPI) network of Khib proteins in *A. niger*. Five clusters of highly interconnected Khib proteins were associated with aminoacyl-tRNA biosynthesis, ribosome, glycolysis/gluconeogenesis, RNA transport and proteasome. These clusters were indicated by different colors and circled black dotted.

We constructed the PPI network of Khib proteins in *A. niger* to investigate how the identified proteins associated with one another in multiple molecular processes. The STRING database was used to establish the PPI network (Figure 5 and Supplementary Table 10). A total of 223 Khib proteins were included in the PPI network, thus, suggesting that Khib proteins are associated with a large range of protein interactions and diverse pathways in the life processes of *A. niger*. They were mainly associated with five highly interconnected clusters: aminoacyl-tRNA biosynthesis, ribosome, glycolysis/gluconeogenesis, RNA transport and proteasome. These results were corresponded to the GO and KEGG pathway enrichment results, and indicated that the identified proteins regulate diverse pathways in *A. niger*.

### Analysis of Khib Proteins Involved in Pathogenesis in *A. niger*

**FIGURE 6:**
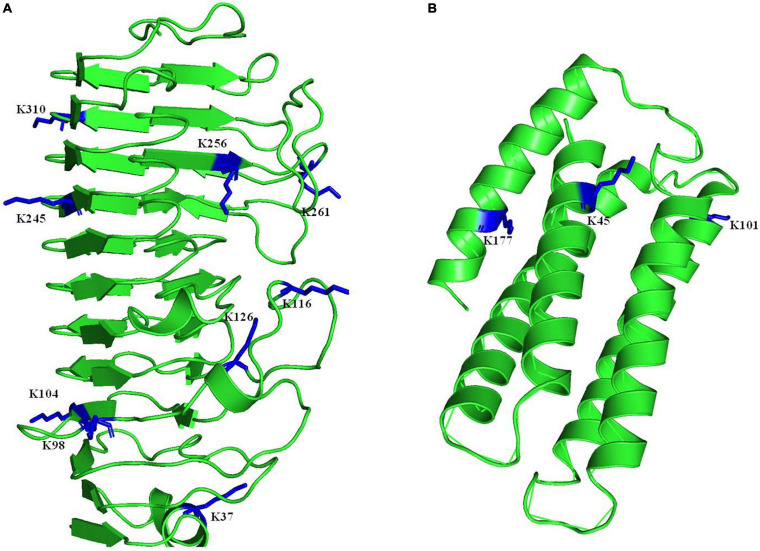
Three-dimensional structure of two proteins that involved in pathogenesis in *A. niger* with identified Khib site. **(A)** The polygalacturonase activity enzyme. **(B)** The Glycoside hydrolases enzyme. The structure was taken from PDB database. The identified sites were highlighted in blue.

Based on the data, we found that several proteins involved in pathogenesis regulation in *A. niger* were 2-hydroxyisobutyrylated (Table 1). Cystathionine beta-synthase (A0A254U7M6) enhances the higher pathogenicity of *A. niger* (Guo et al., 2019), and is located in the cytoplasm and is involved in multiple categories, including sulfur amino acid metabolic process, sulfur amino acid biosynthetic process, organic acid metabolic process. Catalase-peroxidase (A0A254TYP4) is widely found in microorganisms. When the catalase-peroxidase-encoding gene is deleted, the development of lesions is significantly decreased (Zhang et al., 2018). The fungal specific transcription factor domain family protein (A0A254U9C6) encoding endo-1,4-beta-xylanase, facilitates the fungal lifestyle by degrading plant cell wall polysaccharides (Xing et al., 2013). The CLN3 family protein (A0A254TVQ4) and NmrA-like family protein (A0A3F3RAK6) are involved in polygalacturonase enzyme activity in fungal pathogens, which degrades plant cell walls (Liu et al., 2017). Salicylic acid plays a critical role in the plant immune response, so its degradation is therefore important for plant-pathogenic fungi, salicylic acid hydroxylase (A0A254U139) and salicylic acid hydroxylase (A0A254U660) could degrade salicylic acid (Lubbers et al., 2021). These proteins usually have multiple modification sites, such as the Glycoside hydrolases enzyme has 3 modification sites (K45, K101, and K177), the polygalacturonase activity enzyme has 9 modification sites (K37, K98, K104, K116, K126, K245, K256, K261, and K310) (Figure 6). The MS spectra of these two proteins were showed in Supplementary Table 11. Description of the above indicated that Khib proteins may be also involved in the regulation of *A. niger* pathogenicity.

**TABLE 1:** List of identified Khib proteins involved in pathogenesis in *A. niger*.

**Protein accession**	**Name**	**Annotation**	**References**
A0A254U7M6	Cystathionine beta-synthase	Cystathionine beta-synthase and related enzymes	Guo et al., 2019
A0A254TYP4	Catalase-peroxidase	Peroxidase	Zhang et al., 2018
A0A254U9C6	Fungal specific transcription factor domain family protein	Glycoside hydrolases	Xing et al., 2013
A0A254TVQ4	CLN3 family protein	Polygalacturonase activity	Liu et al., 2017
A0A3F3RAK6	NmrA-like family protein	Polygalacturonase activity	Liu et al., 2017
A0A254U139	FAD_binding_3 domain-containing protein	Salicylic acid hydroxylase	Lubbers et al., 2021
A0A254U660	Sugar (And other) transporter family protein	Salicylic acid hydroxylase	Lubbers et al., 2021

## Discussion

Khib is a newly discovered PTM reported in several species, such as mammals, plants, yeast, bacteria and fungi (Huang et al., 2017, 2018; Meng et al., 2017; Yin et al., 2019; Xu et al., 2020). In this research, Khib proteins were studied with proteome-wide analysis. There were 5,041 Khib sites on 1,453 modified proteins in *A. niger* identified from three biological repeats, accounting for approximately 10% proportions of the *A. niger* proteome. Therefore Khib is widely distributed in *A. niger*. The lengths of the modified peptides varied between 7 and 20 aa. The peak of modified peptides was 9aa, and these approximately 1,000 peptides accounted for approximately 12% of all peptides. The peptide length and distribution were similar to those in previous research (Yu et al., 2017). Amino acid motif analysis showed that the substrate preference of Khib in *A. niger*, there were 16 conserved motifs were detected, such as EKhib and KhibK, the negatively charged amino acids D (Aspartic acid) and E (Glutamic acid) showed strong bias around the positions of Khib in *A. niger*, similar to findings in other species including mammals, plants and plant pathogens (Meng et al., 2017; Yu et al., 2017; Huang et al., 2018; Qian et al., 2021). These findings indicated that the positions of the lysines in amino acid sequences play major roles in the modification of Khib. Secondary structure analysis showed that Khib sites were located in different protein secondary structures, thus, suggesting that Khib modifications are widely distributed in all types of proteins.

Khib has been researched in several species, and the conservation analysis of identified proteins from five species showed that *A. niger* has both conserved proteins and newly identified proteins. More than half the identified proteins were found in four species and were identified as completely or well conserved proteins. These data suggested that different species may have unique 2-hydroxyisobutyrylated proteins to perform particular physiological functions, in addition to having large amounts of conserved 2-hydroxyisobutyrylated proteins, which are involved in similar regulation pathways and cellular processes.

The COG/KOG categories showed that 2-hydroxyisobutyrylated proteins are involved in various biological processes, including RNA processing and modification, energy production and conversion, amino acid transport and metabolism, translation, ribosomal structure and biogenesis and post-translational modification, protein turnover and chaperones (Supplementary Figure 3). Functional annotation indicated that 2-hydroxyisobutyrylated proteins were widely distributed in cellular component, molecular function and biological process categories. GO and KEGG pathway enrichment analysis showed that many modified proteins were distributed in categories including ribosome, proteasome, oxidative phosphorylation and citrate cycle (TCA cycle). These pathways are very important for the life processes of *A. niger.* The PPI network indicated five mainly highly interconnected clusters, which showed complicated interaction networks through direct and indirect cooperation and association of various biological processes. In addition, the above results corresponded to one another to a large extent.

In plant pathogens, some PTMs play crucial roles in life processes and pathogenesis, and network analysis of the modified proteins suggested that many extensively studied virulence factors were involved in the highly interconnected protein network. The virulence of plant pathogens have been reported to be partially controlled by epigenetic mechanisms, such as DNA methylation and histone modifications, because these modifications change protein structure and function; therefore, the fungal development and pathogenicity also could be regulated for fungal survival during infection. PTMs modulate not only protein production and activity but also the host response (Dubey and Jeon, 2017; Kong et al., 2018; Nai et al., 2020; Retanal et al., 2021). Khib has recently been reported in *F. oxysporum*, *B. cinerea*, and *U. virens*. In *U. virens*, disruption of 2-hydroxyisobutyrylation in the MAPK pathway kinase UvSlt2 by mutation decreases fungal virulence (Chen et al., 2021). In the comprehensive analysis of *F. oxysporum*, critical proteins for virulence – FoFmk1, FoHog1 and two MAP kinase signaling proteins – have been identified (Qian et al., 2021); For *B. cinerea*, many 2-hydroxyisobutyrylated proteins are involved in substance synthesis and metabolism, redox and autophagy, kinase and protease, and are associated with the pathogenicity of *B. cinerea* (Xu et al., 2020). However, similar studies on the peanut pathogen *A. niger* had not been reported. Our data revealed that Khib is involved not only in the basic life functions of *A. niger* but also in its pathogenic process. As with any other fungus, *A. niger* must degrade the cell wall and overcome plant defense compounds, which have broad antimicrobial properties, in order to successfully infect and colonize a host (Westrick et al., 2021), our research showed that some 2-hydroxyisobutyrylated proteins are associated with polygalacturonase activity and glycoside hydrolases, they usually have more than one Khib sites (Figure 6). These proteins are responsible for fungal decomposition of pectin in plant cell walls and provide the carbon sources for fungal growth (Have et al., 2003; Reignault et al., 2008). Some identified proteins belonged to catalase-peroxidases, which protect fungal pathogens from damage due to the rapid accumulation of reactive oxygen. This rapid accumulation caused by pathogens can amplify the signal quickly and cause cell death in infected plant parts; the pathogen is then restricted to a limited range to prevent its spread (Tondo et al., 2010; Yu et al., 2016). In addition to the pathogenicity-related proteins of *A. niger* that have been reported, many proteins, such as heat shock protein families, are associated with pathogenicity in other species (Cowen et al., 2009; Robbins et al., 2012), and their stress functions have been identified. In the data on Khib proteins in *A. niger*, Hsp110, Hsp90, Hsp70, and Hsp60 are modified; thus, Khib may play an important role in the function of the heat shock protein families. All these identified proteins associated with the virulence of *A. niger* may be regulated by Khib and involved in the network responding to adverse reactions from the host. These results provide new insight into searching for new antifungal methods.

In conclusion, we identified a total of 8,471 sites on 2,030 proteins, including 1,453 proteins with 5,041 identical modification sites in three replicates, thus, providing a large dataset of Khib in fungi. Bioinformatics analysis showed that these modified proteins are not only involved in a broad range of important biological functions and metabolic processes in *A. niger* but also may play an important role in host infection and the response to adverse environmental factors. The global map of 2-hydroxyisobutyrylation in *A. niger* provides a valuable resource to better understand how they perform the biological processes of infecting and harming hosts. The map also provides new ideas to search for new targets to better control the disease.

## Data Availability Statement

The mass spectrometry proteomics data have been deposited in the ProteomeXchange, data are available via ProteomeXchange with identifier PXD026373.

## Author Contributions

MX and YC designed and carried out the experiments, collected and organized the data, and wrote the initial draft of the manuscript. XZ, JY, ZG, YL, XS, KH, and GL participated in designing and carrying out the experiments and reviewed the manuscript. All authors contributed to the article and approved the submitted version.

## Conflict of Interest

The authors declare that the research was conducted in the absence of any commercial or financial relationships that could be construed as a potential conflict of interest.

## Publisher’s Note

All claims expressed in this article are solely those of the authors and do not necessarily represent those of their affiliated organizations, or those of the publisher, the editors and the reviewers. Any product that may be evaluated in this article, or claim that may be made by its manufacturer, is not guaranteed or endorsed by the publisher.

## Footnotes

1
http://www.ebi.ac.uk/GOA/

2
http://www.ebi.ac.uk/interpro/

3
http://string-db.org/

4
https://www.ebi.ac.uk/pride
