# Supplementary material for: Proteome-Wide Analysis of Lysine 2-Hydroxyisobutyrylation in Aspergillus niger in Peanuts
Source: Front Microbiol. 2021 Aug 18;12:719337. doi: 10.3389/fmicb.2021.719337 (PMC8418202; doi:10.3389/fmicb.2021.719337)
Supplement: Supplementary file 1 [file Table_1.docx]

**Cover page of supplementary material for** **“Proteome-Wide Analysis of Lysine** **2-Hydroxyisobutyrylation in the** ***Aspergillus niger* in Peanuts”**

**Supplementary Table 1.** Basic analysis of MS identified information.

**Supplementary Table 2**. Annotation combine of identified proteins.

**Supplementary Table 3**. Detailed information of motif annotation of identified proteins.

**Supplementary Table 4**. Detailed information of 2-hydroxyisobutyrylated Lys conserved analysis.

**Supplementary Table 5**. Detailed information of GO terms of level 2 distribution of all proteins.

**Supplementary Table 6**. Detailed information of subcellular localization of the identified protein.

**Supplementary Table 7**. GO enrichment analysis of modified proteins.

**Supplementary Table 8**. Detailed information of KEGG pathway.

**Supplementary Table 9**. Detailed information of protein domain.

**Supplementary Table 10**. Detailed information of PPI network proteins.

**Supplementary Table 11**. The MS spectra of two proteins that involved in pathogenesis.

**Supplementary Figure 1**. Proteome-Wide Analysis of Lysine 2-Hydroxyisobutyrylation sites in the *A. niger*. (A) Overview of experimental procedures used in this research. (B) Peptide length distribution. (C) Peptide mass tolerance distribution.

**Supplementary Figure 2**. Khib proteins were involved in ribosomes and proteasomes. (A) 2-hydroxyisobutyrylated enzymes were involved in ribosomes. (B) 2-hydroxyisobutyrylated enzymes were involved in proteasomes. The identified proteins are highlighted in red.

**Supplementary Figure 3**. Identification by COG KOG categories.
