# Supplementary figures and images for: Proteome-Wide Analysis of Lysine 2-Hydroxyisobutyrylation in Aspergillus niger in Peanuts
Source: Front Microbiol. 2021 Aug 18;12:719337. doi: 10.3389/fmicb.2021.719337 (PMC8418202; doi:10.3389/fmicb.2021.719337)

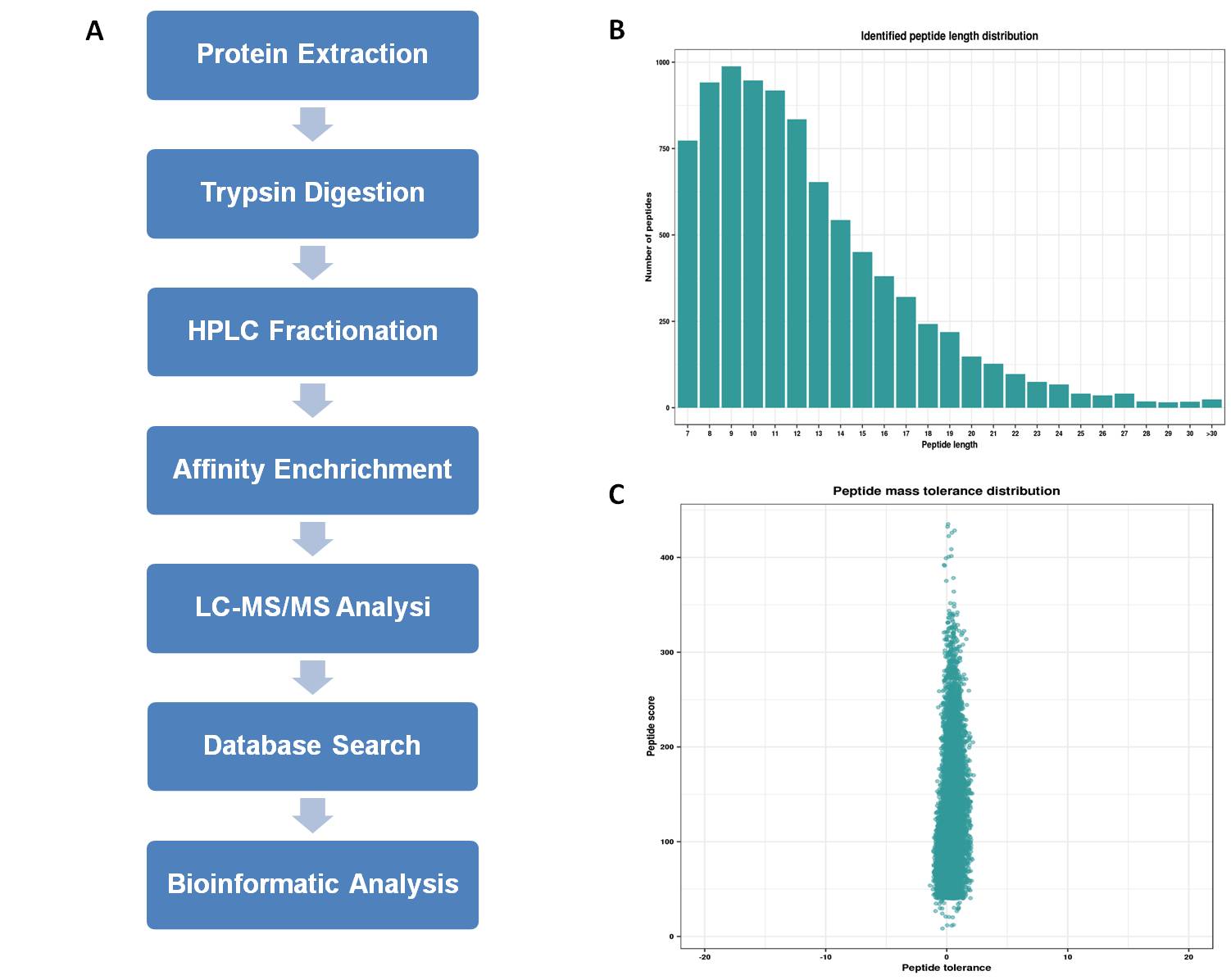

Supplement: Supplementary file 13 [file Image_1.jpg]

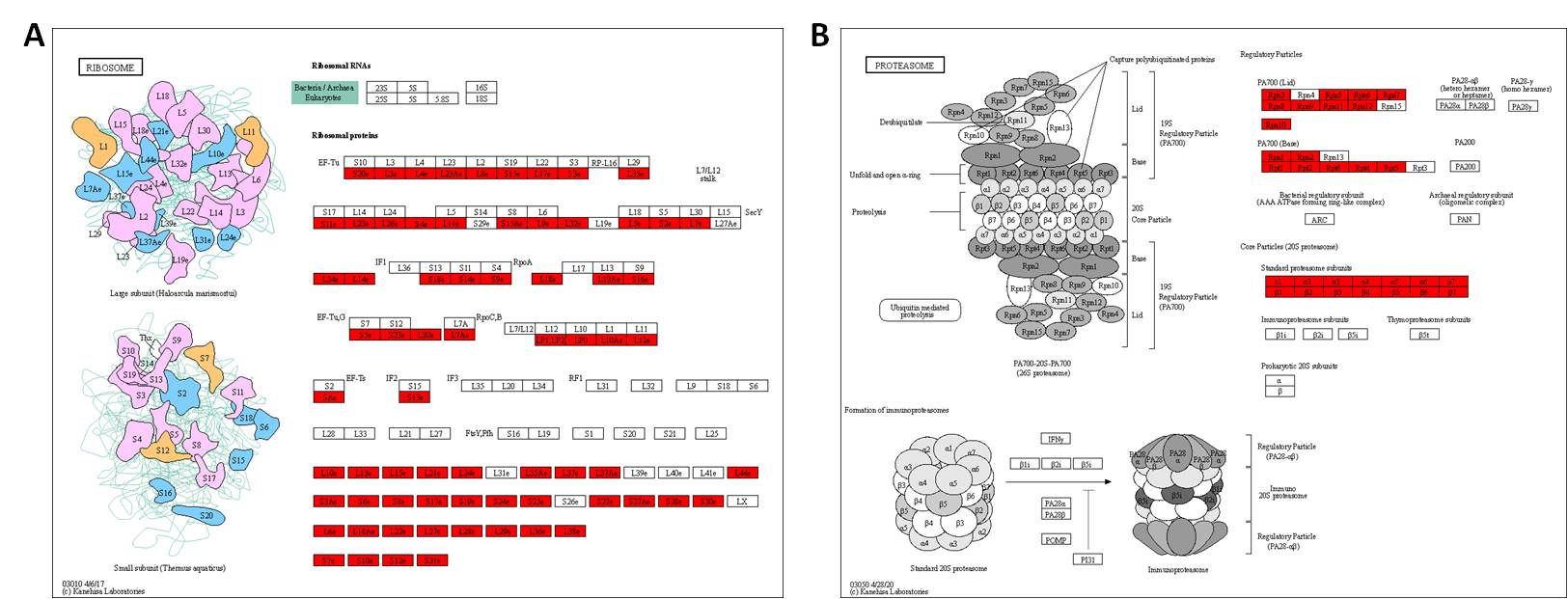

Supplement: Supplementary file 14 [file Image_2.JPEG]

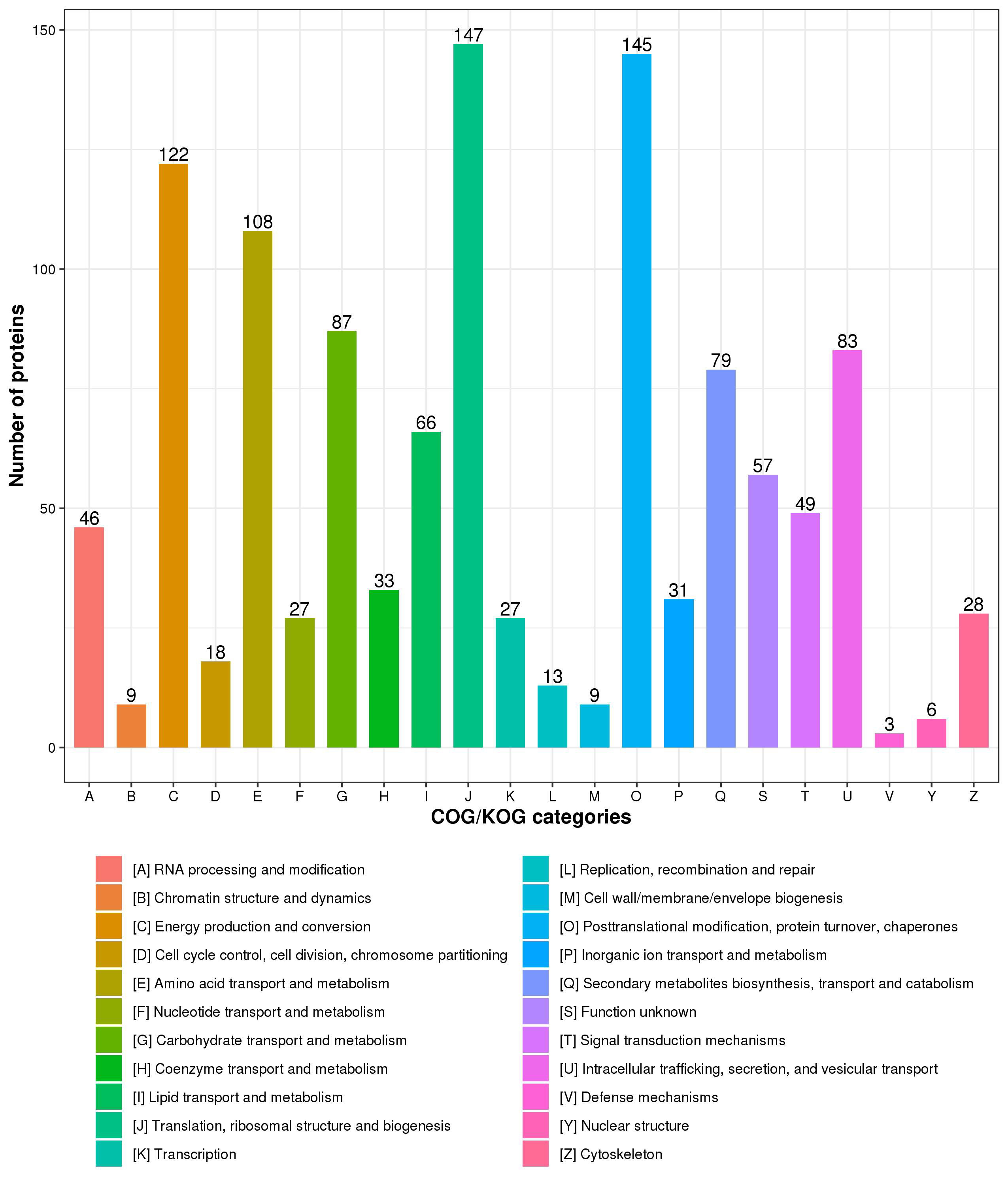

Supplement: Supplementary file 15 [file Image_3.JPEG]
